# The ability of natural tolerance to be applied to allogeneic tissue: determinants and limits

**DOI:** 10.1186/1745-6150-2-10

**Published:** 2007-04-16

**Authors:** William FN Chan, Ainhoa Perez-Diez, Haide Razavy, Colin C Anderson

**Affiliations:** 1 Department of Surgery, Surgical-Medical Research Institute, University of Alberta, Edmonton, Alberta, Canada; 2 Department of Medical Microbiology and Immunology, Surgical-Medical Research Institute, University of Alberta, Edmonton, Alberta, Canada; 3 Ghost Lab, Laboratory of Cellular and Molecular Immunology, National Institute of Allergy and Infectious Diseases, National Institutes of Health, Bethesda, Maryland, USA

## Abstract

**Background:**

Transplant rejection has been considered to occur primarily because donor antigens are not present during the development of the recipient's immune system to induce tolerance. Thus, transplantation prior to recipient immune system development (pre-immunocompetence transplants) should induce natural tolerance to the donor. Surprisingly, tolerance was often not the outcome in such 'natural tolerance models'. We explored the ability of natural tolerance to prevent immune responses to alloantigens, and the reasons for the disparate outcomes of pre-immunocompetence transplants.

**Results:**

We found that internal transplants mismatched for a single minor-H antigen and 'healed-in' before immune system development were not ignored but instead induced natural tolerance. In contrast, multiple minor-H or MHC mismatched transplants did not consistently induce natural tolerance unless they carried chimerism generating passenger lymphocytes. To determine whether the systemic nature of passenger lymphocytes was required for their tolerizing capacity, we generated a model of localized vs. systemic donor lymphocytes. We identified the peritoneal cavity as a site that protects allogeneic lymphocytes from killing by NK cells, and found that systemic chimerism, but not chimerism restricted to the peritoneum, was capable of generating natural tolerance.

**Conclusion:**

These data provide an explanation for the variable results with pre-immunocompetence transplants and suggest that natural tolerance to transplants is governed by the systemic vs. localized nature of donor antigen, the site of transplantation, and the antigenic disparity. Furthermore, in the absence of systemic lymphocyte chimerism the capacity to establish natural tolerance to allogeneic tissue appears strikingly limited.

**Reviewers:**

This article was reviewed by Matthias von Herrath, Irun Cohen, and Wei-Ping Min (nominated by David Scott).

## Open peer review

Reviewed by Matthias von Herrath, Irun Cohen, and Wei-Ping Min (nominated by David Scott). For the full reviews, please go to the Reviewers' comments section.

## Background

Transplantation of donor cells/tissues prior to the development of recipient immunocompetence theoretically provides the greatest opportunity to achieve donor specific tolerance (for our definition of key terms, such as tolerance, see Additional File 1). All of the tolerance processes that occur for self-reactive T cells are potentially available for donor reactive T cells, and in most cases the transplant has time to heal-in prior to encountering the recipient's immune system, potentially eliminating or reducing the APC activating signals from damaged tissues [1-3]. The observations of Owen and Medawar and colleagues [4-6], together with the theories of Burnet and Fenner [7], led to the generally held view that allogeneic donor cells or tissue given before immunocompetence should be treated as self tissue and induce tolerance [8-11]. However, over the last 30 years the data are almost equally divided been support and rejection of this view. For example, Owen described a natural situation where dizygotic cattle twins possessed blood cells of their fraternal twin and remained life long chimeras, appearing to treat the foreign cells as self [4]. Experiments by Medawar's group [6], and also those of Hasek [12], showed that experimentally introducing foreign blood cells early in life (before full immunocompetence) could lead to tolerance of donor antigens. The B cell tolerance of ABO incompatibilities in infant cardiac transplantation is a dramatically successful recent application of the pre-immunocompetence graft concept [10,13]. In contrast, studies by Le Douarin and colleagues showed that xenogeneic and allogeneic limb buds grafted into embryos were not treated as self by the newly generating immune system [14,15]. Similarly, McCullagh showed that pre-immuncompetence allogeneic fetal skin grafts given to fetal sheep could trigger immunity rather than tolerance [16]. More recently other natural tolerance models have been examined. In these models, grafts are given pre-immunocompetence by using genetically immunodeficient adult recipients and allowing T cells to develop de novo in the presence of the transplant. Like the studies of Le Douarin and McCullagh, these studies showed that allogeneic grafts (skin, heart or islets) given pre-immunocompetence were not treated as self but instead triggered immunity [2,3,17-20]. We hypothesize that the paradox of immunity or tolerance with pre-immunocompetence grafts is most simply explained by the nature and distribution of the tissues involved (donor tissue and host site), and the length of time the grafts are established before immunocompetence sets in. Experiments that showed tolerance used hematopoietic cells as the donor tissue while those showing immunity used solid tissue grafts. Hematopoietic cells can migrate systemically raising the possibility that solid tissue grafts fail because their antigens remain localized in the periphery and the natural peripheral tolerance mechanisms are unable to act on the alloreactive T cell repertoire encountering antigens that are not widely distributed. Consistent with this hypothesis, we found that pre-immunocompetence single minor-H mismatched skin grafts only induced tolerance if they contained passenger lymphocytes capable of generating systemic chimerism [20]. However, at least two important questions remain unanswered. 1. Are natural tolerance mechanisms ever able to successfully induce tolerance to antigens of allogeneic tissue without systemic lymphocyte chimerism and if so what determines this outcome? 2. Are hematopoietic cells better able to take advantage of natural tolerance mechanisms because they migrate systemically or would they also be better at inducing tolerance when present in a locally restricted fashion? Answering these questions is critical to understanding the potential and limits in the capacity of natural tolerance to act on allogeneic tissue. Herein we tested whether the degree of antigenic mismatch and the type of donor tissue and its distribution determine the ability of natural tolerance to be established to alloantigens.

## Results

### Natural tolerance can be established to minimally mismatched internal transplants

**Figure 1:**
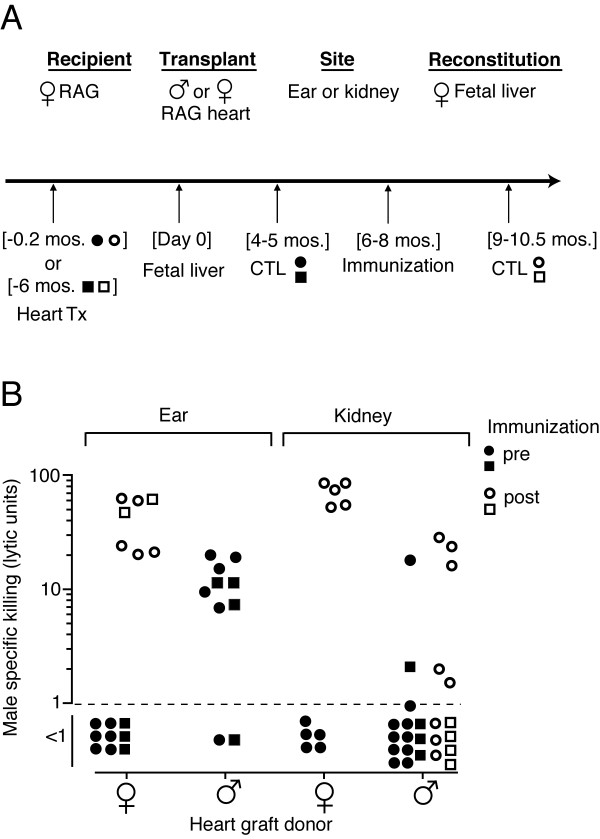
Pre-immunocompetence single minor-H mismatched transplants are not ignored but instead trigger immunity or natural tolerance depending on the graft site. (A) Experimental design and timeline; (B) Male specific killing for spleen cells from individual mice is depicted. B10-RAG female recipients were given a B10-RAG male or female cardiac graft under the kidney capsule or under the skin of the ear pinna. Less than 10 days (circles) or 6 mos. (squares) later these mice were reconstituted with fetal liver cells from female B6 fetuses. 4–5 mos. post reconstitution they were hemisplenectomized and tested for CTL priming to the H-Y antigen (1^st ^CTL; solid symbols; Male heart vs. female heart: in the ear, p < 0.001, and in the kidney, p > 0.05). Some of the mice were later immunized i.p. with 3 × 10^6 ^B6 male spleen cells and 6 or more wks after immunization (9–10.5 mos. after FL) they were tested again for CTL priming to the H-Y antigen (2^nd ^CTL; open symbols; for grafts in the kidney: pre- vs. post-immunization, female heart, p < 0.001, male heart, p > 0.05; post-immunization male vs. female heart, p < 0.01).

Contrary to the expectations of theories postulating an early tolerance window, solid tissue transplants given pre-immunocompetence generally trigger immunity rather than tolerance, even in the case of skin grafts with the minimal H-Y mismatch [21]. These skin transplants only induced tolerance if they carried passenger T cells capable of generating systemic chimerism [20]. We examined whether the inability to induce natural peripheral tolerance to an allogeneic tissue was absolute or if instead tolerance might be established to weakly mismatched internal transplants; the exposure of skin grafts to the external environment, or other factors may reduce their ability to establish peripheral tolerance. Since an H-Y mismatch alone is not sufficient to trigger rejection of tissues other than skin grafts, our question was instead whether the pre-immunocompetence male internal transplant would be immunologically ignored [22] or induce tolerance. The general experimental design (Figure 1A) was to give female B6-RAG immunodeficient recipients male transplants prior to recipient adaptive immune system generation, the latter being achieved by injecting wild type (WT) female fetal liver (FL) cells containing hematopoietic stem cells. The male donors were RAG in order to eliminate systemic chimerism by passenger lymphocytes [20]. In order to keep the graft tissue constant and vary its location, we used a non-vascularized graft model. We placed neonatal male heart tissue either under the skin of the ear or internally under the kidney capsule of B6-RAG female recipients. Grafts were allowed to heal in for a short period (<10 days) or for 6 months prior to fetal liver reconstitution. Reconstituted recipients were hemisplenectomized and tested for CTL priming to HY. As unprimed mice do not generate CTL *in vitro *to minor antigens, this was a test for prior *in vivo *priming. Similar to the CTL priming that is seen in recipients of a male skin graft [20], most of those mice that received the male heart graft under the skin, were primed by the graft and made anti-HY CTL (control female grafts did not stimulate a CTL response to HY). In contrast, almost all mice that received the graft under the kidney capsule showed no response (Figure 1B). We then asked whether the lack of *in vivo *CTL priming in recipients with a graft under the kidney capsule was due to ignorance of the graft antigen or whether the long-term presence of the un-rejected graft might induce tolerance to the H-Y graft antigen. We immunized the recipients with male spleen cells and a month or more later, assessed whether the mice were primed for an anti-HY CTL response. Control mice with female heart grafts responded well to the immunization while the majority of recipients of a male graft under the kidney capsule did not respond or responded weakly (Figure 1B); interestingly, those that did make some response were in the short healing time group. To begin to assess whether a different tissue transplanted internally would also induce tolerance, we gave diabetic B6-RAG female recipients a B6-RAG male islet transplant followed later by fetal liver cell reconstitution. A preliminary analysis indicated T cells that developed in these recipients were also specifically tolerant rather than ignorant of H-Y (See Additional File 2). Together these data suggested that an internal weakly mismatched transplant is not ignored by a newly generating immune system, instead the location of the established graft determines whether the graft triggers CTL priming or tolerance.

### Low frequency of natural tolerance with multiple minor mismatches; requirements for chimerism

**Figure 2:**
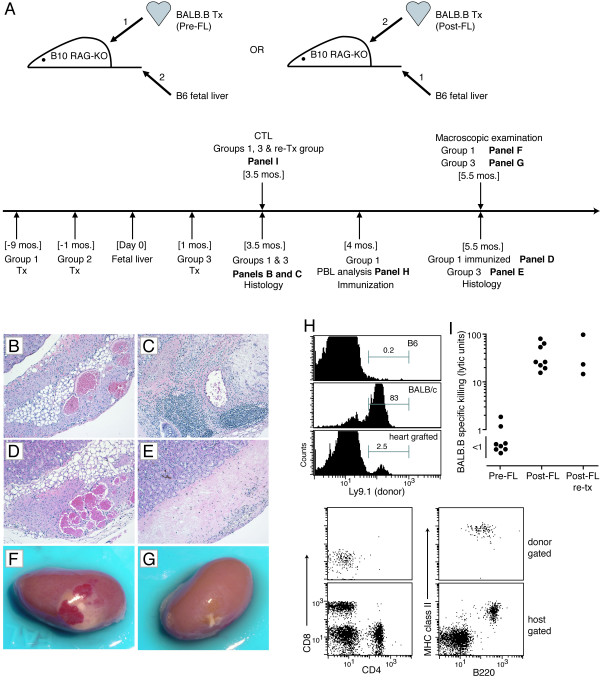
Natural tolerance to multiple minor internal transplants carrying passenger lymphocytes. (A) Experimental design and timeline. BALB.B heart grafts were given under the kidney capsule of B10-RAG recipients 9 or 1 mos. before (pre-FL) or 1 mos. after (post-FL) they were reconstituted with B6 fetal liver cells. Visual inspection of grafts at 3.5 mos. after FL injection showed all 9 mos. healed grafts (n = 8) were present and beating, while 7 of 8 grafts given post-FL and all those healed only 1 mos. (n = 4) were completely gone (only scar tissue remaining). Some recipients with a 9 mos. healed in graft were immunized with 3 × 10^6 ^BALB.B spleen cells 4 mos. after FL injection (n = 3) and others received a second BALB.B heart graft under the capsule of the opposite kidney 4.5 mos. post-FL (n = 4); these second BALB.B cardiac grafts were all present and beating at 3.5 months post transplantation (8 mos. after FL). (B-G) Representative macroscopic and histological (100X) analyses of BALB.B cardiac grafts. (B) Histology of a 9 mos. healed in pre-FL BALB.B cardiac graft under kidney capsule at 3.5 mos. post-FL reconstitution. (C) Histology of the 1 out of 8 post-FL BALB.B heart grafts that had not been completely rejected at 3.5 mos. post-FL cell reconstitution; the graft was not beating. (D) Histology of a 9 mos. healed in pre-FL BALB.B cardiac graft under kidney capsule at 5.5 mos. post-FL cell reconstitution and 5 weeks post immunization with BALB.B spleen cells. (E) Histology of a post-FL BALB.B cardiac graft under kidney capsule at 5.5 mos. post-FL cell reconstitution (only fibrotic tissue remains). (F) Representative appearance of cardiac grafts that are not rejected (beating); a 9 mos. healed in pre-FL BALB.B cardiac graft under kidney capsule (5.5 mos. post-FL cell reconstitution and 5 wks. post immunization with BALB.B spleen cells) is shown. (G) Representative appearance of rejected cardiac grafts; what remains (scar tissue) of a post-FL BALB.B cardiac graft under kidney capsule (5.5 mos. post-FL cell reconstitution) is shown. (H) Staining for donor (Ly9.1^+^) BALB.B cells in PBL of B10-RAG recipients of a BALB.B heart graft (9 mos. healed; lower histogram) 4 mos. following reconstitution with B6 FL cells and in control B6 and BALB/c mice; at the same time, four color flow cytometry for B and T cells was done (gated on donor (Ly9.1^+^) vs. host (Ly9.1^-^) cells) in the heart graft recipients (representative dot plots are shown). (I) Donor specific CTL in individual B10-RAG mice, reconstituted 3.5 mos. previously with B6 FL cells, that had received a BALB.B heart graft 9 mos. pre-FL or post-FL (p < 0.01). In a third group the ability of the long established heart graft to prime CTL was tested by re-transplanting 9 mos. established BALB.B hearts from B10-RAG recipients into B10-RAG mice previously reconstituted with B6 FL (Post-FL, re-tx; p < 0.05 vs. pre-FL).

**Figure 3:**
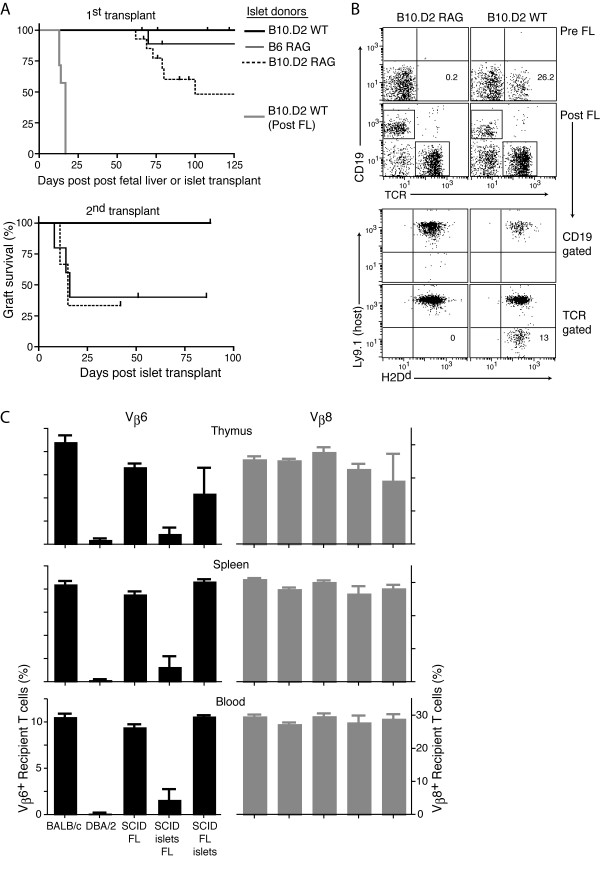
Reduced natural tolerance to increasingly mismatched internal transplants lacking passenger lymphocytes; requirements for systemic chimerism. (A) Top survival graph, 1^st ^transplant: Graft survival in days post fetal liver injection is shown for STZ induced diabetic BALB/c-SCID mice that received B10.D2 WT vs. B10.D2-RAG islets (n = 11 and 14 respectively; p = 0.009) or B6-RAG islets (n = 10; p = 0.01 vs. B10.D2-RAG) 4–7 months pre-FL; control syngeneic BALB/c islets (not shown) survived long-term post FL reconstitution (>131, >185 × 3 days). Also shown is graft survival in days post islet transplant of B10.D2 WT islets (n = 7) given >13 weeks post-FL (thick grey line). Some recipients with functioning grafts beyond 100 days were given a 2^nd ^donor islet transplant (lower survival graph; B10.D2 WT, n = 8, vs. B10.D2 RAG, n = 3, p = 0.011; B6 RAG, n = 5). (B) Representative flow cytometry analyzing the presence and phenotype of donor cells from the PBL of mice shown in (A). T cells of the donor (Ly9.1^-^) are present in recipients of WT but not RAG islets (lower right vs. left dot plots respectively). All 11 recipients were T cell chimeras, with 3.2 ± 1.6% of T cells being of donor origin (Ly9.1^- ^TCR^+^) in 7 mice analyzed at 30 weeks post FL injection. (C) Frequency of donor reactive (Vβ6) and control (Vβ8) T cells in the thymus (gated on host CD4 SP cells), spleen and blood (gated on host TCR^+ ^cells) of normal BALB/c and DBA/2 mice, and BALB/c-SCID mice given BALB/c FL alone or DBA/2 islets before or after the FL (analysis at >33 wks after FL injection; mean ± SE; n = 2–5 mice per group). DBA/2 islets given after FL were rejected ~4 days prior to the Vβ analysis. Chimerism with islet-derived donor T cells (CD5.1^+^) in the spleen of the recipients with islets given pre-FL ranged from 1.4–3.9% of all T cells. Percent Vβ6 comparing SCID FL vs. SCID islets FL: p < 0.001 for thymus, blood, and spleen; comparing SCID islets FL to SCID FL islets: thymus, p > 0.05, spleen and blood, p < 0.001.

Having provided evidence that natural tolerance can be established to antigens in well-healed minimally mismatched allogeneic tissue, we next determined if this could be extended to greater mismatches. We allowed multiple minor-H mismatched BALB.B neonatal heart grafts to heal-in for one or nine-months in B10-RAG recipients prior to B6 fetal liver cell reconstitution (pre-FL). To compare such "healed-in" pre-FL grafts with "fresh" grafts, another age-matched cohort of recipients was given a heart graft one-month after fetal liver cell reconstitution (post-FL). Fourteen-weeks post fetal liver injection the status of the grafts was assessed. All of the nine-month healed grafts were accepted (present and beating) and these recipients appeared tolerant rather than ignorant of the donor as they accepted a second donor heart transplant given post immune reconstitution. Furthermore, immunization with donor spleen cells did not trigger rejection of long established grafts. In contrast, seven out of eight fresh grafts and all one-month healed grafts were completely rejected (Figure 2). In the one post-FL graft where it appeared that some graft tissue remained, the graft was not beating and it was massively infiltrated with lymphocytes when analyzed histologically, unlike the long established heart tissue (Figure 2C vs. 2B). All of those mice that received a fresh heart graft, but not a long-healed graft, were immunized by the graft and made strong anti-BALB.B CTL responses (Figure 2I). The lack of CTL priming in mice with long-healed grafts was not due to a loss of donor antigens, as re-transplantation of the grafts into naïve recipients triggered CTL priming. This was consistent with the apparent tolerance in recipients of long-healed grafts, as demonstrated by a lack of both CTL priming (not shown) and damaging lymphocytic infiltration of the graft after spleen cell immunization (Figure 2D; focal lymphocytes adjacent to the graft were seen in some sections, not shown) as well as acceptance of second donor transplants. While these studies indicated natural tolerance could be established to multiple minor-H mismatched transplants, we had initially assumed that the donor heart tissue, being neonatal, would lack sufficient passenger lymphocytes to generate chimerism and therefore would represent a model of localized donor antigen. However, this assumption was incorrect. Figure 2H shows that recipients of a neonatal BALB.B heart graft contained donor Ly9.1^+ ^cells circulating in their blood and these cells in most animals were donor B cells (MHC class II^+ ^and B220^+^; also CD19^+^, not shown). A preliminary comparison of hearts from WT vs. RAG multiple minor-H mismatched donors suggested that the chimerism generating WT grafts were more able to induce tolerance. To more extensively examine whether natural tolerance to multiple minor-H mismatches requires passenger lymphocyte derived chimerism we compared islet transplants from WT vs. RAG donors on the B10.D2 background given to BALB/c-SCID recipients (Figure 3). Control syngeneic BALB/c islets survived long-term post immune reconstitution, as did all established B10.D2 WT donor islets and these recipients universally accepted a second donor islet transplant (Figure 3A). In contrast, half of the recipients of B10.D2-RAG islets rejected the islet transplant (including grafts healed-in for seven months) and those that did not were often able to reject a second donor transplant. We also assessed the fate of MHC mismatched B6-RAG islets, which were rejected significantly less often than those that differed only by multiple minors. However, the majority of recipients rejected a second donor transplant.

Thus, consistent tolerance to multiple minor-H mismatches was only achieved with WT donors that were able to generate lymphocyte chimerism in the recipients (Figure 3B). Unlike the neonatal heart grafts in the BALB.B to B10-RAG combination, that generated B cell chimerism, the adult B10.D2 WT islets transplanted to BALB/c-SCID recipients generated almost exclusively T cell chimerism in the blood, similar to what we had seen previously with skin grafts [20]. To assess whether chimerism from islet-derived passenger lymphocytes induced tolerance via deletion of newly generated recipient T cells, we transplanted DBA/2 islets into BALB/c-SCID mice, in which the frequency of Vβ6^+ ^recipient T cells that are reactive to donor superantigens can be assessed. Recipient Vβ6^+ ^but not control Vβ8^+ ^T cells were greatly reduced in frequency in recipients of DBA/2 islets compared to controls without an islet transplant. The loss of Vβ6^+ ^T cells was evident not only in the periphery but also in the thymus and occurred when the islets were given prior to but not after immune reconstitution (Figure 3C).

### Recipient natural killer cells restrict donor cell chimerism to the peritoneal cavity: natural tolerance of systemic but not locally restricted donor cells

**Figure 4:**
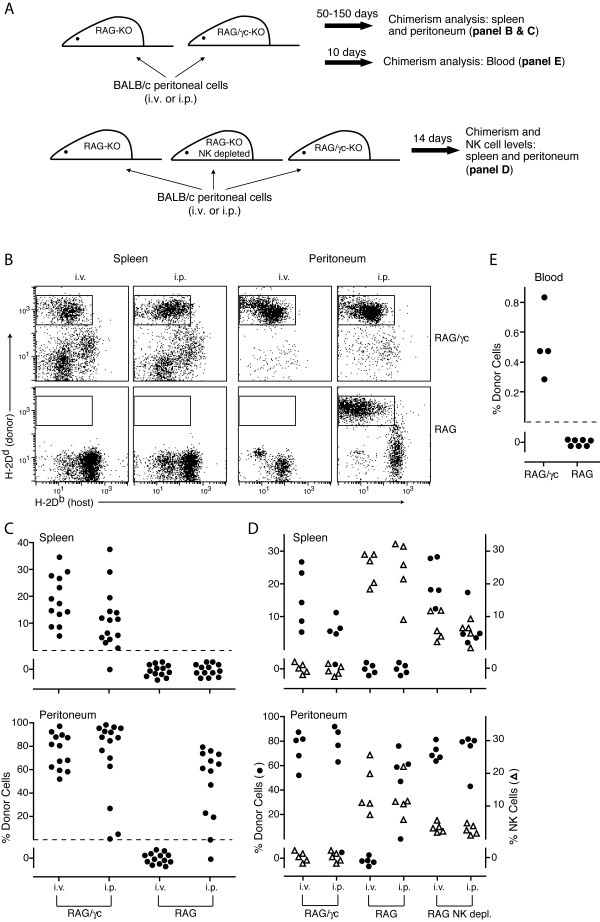
Donor lymphocytes are restricted to the peritoneal cavity in NK sufficient but not NK deficient recipients. (A) Experimental design. (B, C) Chimerism assessed from 50–150 days post i.v. vs. i.p. injection of donor BALB/c peritoneal cells into RAG vs. RAG/γc mice. (B) Representative flow cytometry data assessing donor cell chimerism in the lymphocyte gate of recipient spleen and peritoneal cells. Donor cells are defined by the box in each dot plot. (C) The percentage of donor cells in the recipient spleen and peritoneum. Each symbol represents values for an individual mouse. (D) The percentage of donor cells and NK cells in the spleen and peritoneum of RAG/γc, RAG, and anti-asialo GM1 treated RAG mice 2 weeks post injection, i.v. or i.p., of BALB/c peritoneal cells. (E) Chimerism in peripheral blood of individual recipients assessed 10 days post i.p. injection of donor BALB/c peritoneal cells into RAG vs. RAG/γc mice.

Having established that natural tolerance to donor cells/tissue does not readily occur in the absence of passenger lymphocytes, with the notable exception of minimal minor-H mismatches, the question then arose as to why donor lymphocytes were better tolerogens than solid donor tissues or cell aggregates (e.g. islets). A major difference between such solid tissues and donor lymphocytes is the localized vs. systemic nature of donor antigen respectively (donor lymphocytes migrate). This difference is often assumed to be critical in explaining the tolerizing ability of donor lymphocyte infusions. However, to our knowledge this assumption has never been tested. We set up a system where donor lymphocytes, present before recipient immune system generation, were restricted in location vs. systemic and asked if they were equally capable of inducing tolerance (see experimental design in Figure 4A). We made use of a serendipitous observation that MHC mismatched lymphocytes cannot escape or survive outside the peritoneal cavity when the recipient has functional NK cells. We chose peritoneal cells, depleted of most T cells (to avoid GVHD), as a source of donor lymphocytes because they contain a large population of self-sustaining lymphocytes, the B-1 cells (C19^+^CD43^+^CD5^+^) [23], and because of the tolerance observed in recipients with long lasting passenger B cell chimerism (Figure 2H). BALB/c peritoneal cells were injected into NK cell sufficient B6-RAG mice or NK cell deficient B10-RAG/γc mice. As anticipated, the allogeneic donor lymphocytes injected either i.v. or i.p. into B10-RAG/γc mice survived long-term and were present at high frequency in the spleen and peritoneum (Figure 4B, C). In sharp contrast, donor cells did not survive when injected i.v. into B6-RAG mice. However, when donor cells were injected i.p. into B6-RAG mice they were able to survive in the peritoneum but were not found in lymphoid tissue outside the peritoneum, including the spleen, lymph nodes, bone marrow and thymus (Figure 4 and data not shown). These differences between RAG vs. RAG/γc recipients were also observed by assessing donor cells in peripheral blood; donor cells were present at low levels in all RAG/γc mice but in none of the RAG recipients (Figure 4E). These data suggested that, once in circulation, the donor cells were susceptible to killing by NK cells and that the peritoneum is instead a 'protected zone' where the donor cells can either resist NK cell killing, or perhaps do not stimulate NK activity. To further examine whether NK cells were at the root of the differing results in RAG vs. RAG/γc mice, we compared the ability to establish donor cell chimerism in RAG mice and RAG mice depleted of NK cells. Figure 4D shows that NK cell depletion of RAG mice allowed chimerism to be established using the i.v. route of injection, and allowed donor cells to migrate and survive outside the peritoneum (in the spleen) when injected i.p.

**Figure 5:**
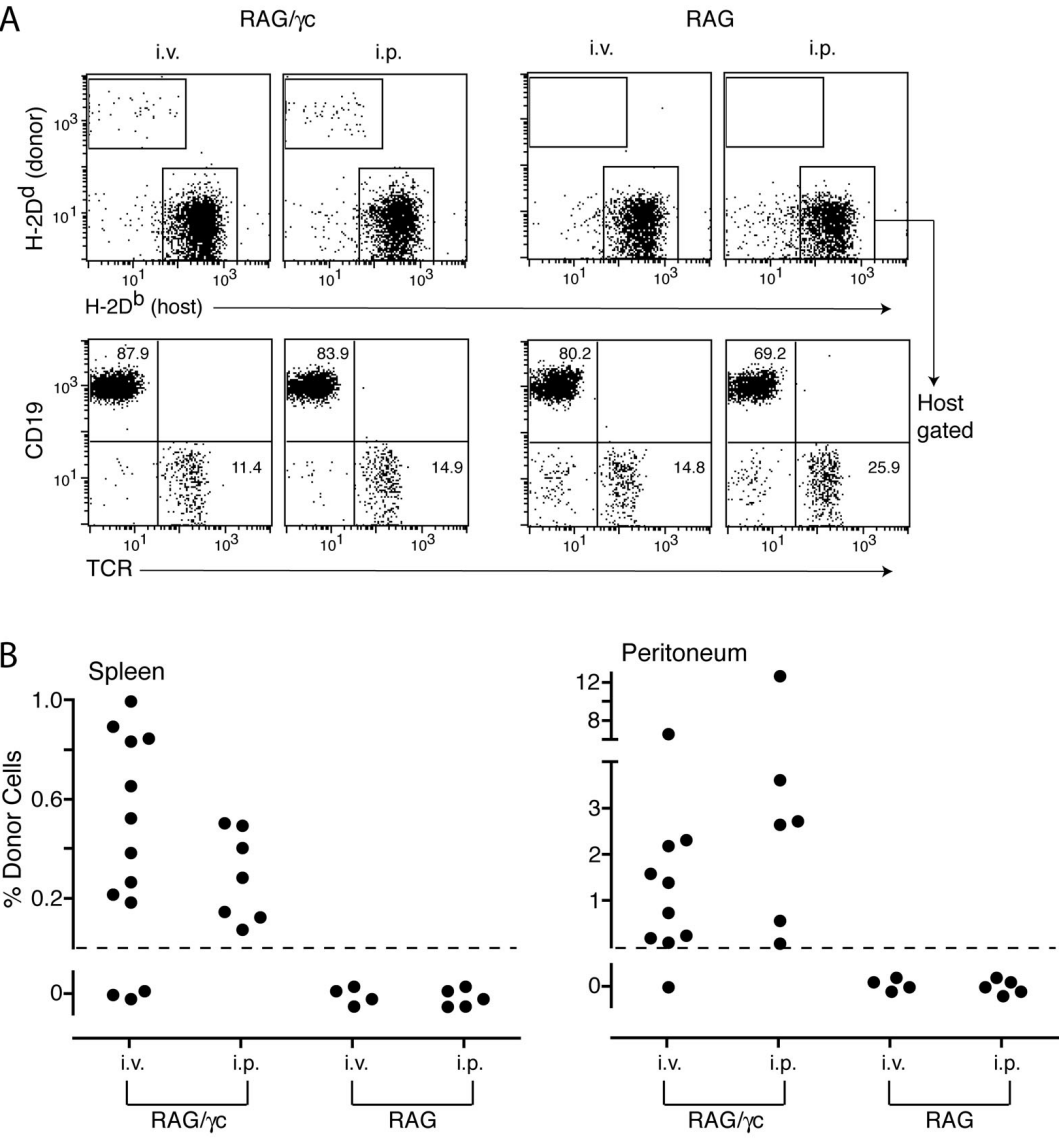
Natural tolerance of donor lymphocytes when present systemically but not when restricted to a peripheral site. Donor cell chimerism assessed from 50–150 days post B6 fetal liver immune reconstitution of RAG vs. RAG/γc mice previously injected i.v. vs. i.p. with donor BALB/c peritoneal cells (as described in Figure 4A). (A) Representative flow cytometry data assessing donor cell chimerism amongst recipient peritoneal cells. Donor and recipient cells are defined by the upper left and lower right box in each top row dot plot respectively. Lower dot plots show recipient B and T cell reconstitution after gating on host cells as shown in the lower right box of the upper dot plots. (B) The percentage of donor cells in the spleen and peritoneum of individual recipients. Values for spleen plus peritoneum comparing RAG/γc vs. RAG: i.v. p < 0.01, and i.p. p < 0.001.

Having established local vs. systemic donor lymphocyte chimerism, we asked whether a newly generated immune system tolerates or rejects donor cells when restricted locally to the peritoneum (RAG recipients) vs. when they are present more systemically (RAG/γc recipients). Figure 5 shows that donor cells persisted in both the spleen and peritoneum of most reconstituted RAG/γc mice (though at a lower frequency once the fetal liver cells established an immune system). In contrast, any local chimerism established in the peritoneum of RAG mice failed to persist post immune reconstitution, as donor cells were undetectable in all reconstituted RAG recipients.

## Discussion

Self-tolerance due to encounter of self but not foreign antigens as the immune system develops has long been considered the primary factor that controls immunity vs. tolerance (often termed self non-self discrimination); however, despite its appeal in explaining transplant rejection, this parsimonious solution to the immunity-tolerance problem has encountered its strongest challenge from data in the transplantation field itself (reviewed in [21]). Pre-immunocompetence transplants should theoretically have the same opportunity to trigger natural tolerance mechanisms as do peripheral self-antigens and therefore pharmacologic immunosuppression may not be needed under these conditions. We therefore refer to pre-immunocompetence transplants as natural tolerance models. Given these considerations, it has been surprising that pre-immunocompetence solid tissue transplants have almost universally generated immunity [2,3,14-20] or putative ignorance [18] rather than tolerance. However, almost all of these studies were done with fully MHC and minor-H antigen mismatched transplants of skin, a strong stimulus that is apparently too strong for natural peripheral tolerance mechanisms to cope with. Nevertheless, reducing the mismatch to as little as the single minor-H antigen H-Y was not sufficient to establish natural tolerance to a pre-immunocompetence skin graft, despite the apparent establishment of full self-tolerance in this model [2]. We therefore examined whether the ability of natural tolerance to take hold with allogeneic tissue may be determined not only by the degree of antigenic mismatch but also by the type of tissue or its location. The data herein indicate that natural tolerance is established to minimally mismatched heart and islet tissue transplanted internally (under the kidney capsule), but in contrast the same heart tissue placed under the skin immunized the recipient to the donor antigen. The ability of graft location to determine immunity vs. natural tolerance to the graft is unlikely to be related to the phenomena of immune privileged sites [24], as the sub-renal capsule location is not immune privileged. Furthermore, when the mismatch was increased to multiple minor or MHC mismatches, such pre-immunocompetence transplants in the kidney were able to induce tolerance only in a small fraction of recipients unless they carried passenger lymphocytes that generated systemic chimerism. The systemic chimeras demonstrated a strong loss of donor superantigen reactive T cells, which likely occurred via crosspresentation of the superantigen [25]. The most obvious conclusion from these studies is that the capacity to establish natural tolerance to allogeneic tissue is quite limited without chimerism, consistent with the potential role of chimerism observed in clinical organ transplant recipients [26]. The identification of the peritoneum as a site where chimeric cells can resist killing by host NK cells allowed us to test whether the differing outcome between pre-immunocompetence hematopoietic and solid tissue transplants is related to the systemic vs. localized nature respectively of the donor cells. While we have not yet identified the reasons behind the protection of donor peritoneal cells from NK cell killing in the peritoneum, our data indicate that donor chimerism restricted to this peripheral site does not induce tolerance in a newly generated immune system. In contrast, pre-immunocompetence systemic chimerism achieved in NK cell deficient recipients survives long after development of the recipient's immune system. These data suggest that, in addition to tolerance in the newly generated host T cell compartment, host NK cells generated from the fetal liver also become tolerant of donor cells. We expect this NK cell tolerance will involve a regulation of activating/inhibitory receptor expression during NK cell development [27], a process that does not readily occur in the already mature NK cell repertoire of the RAG recipients. The mechanism of resistance to NK cell killing by donor cells in the peritoneum could include differences in peritoneal NK cell function or number relative to other sites or an absence of the stress induced changes that trigger NK cells [28-30] when donor peritoneal cells are placed in their natural environment.

Together with recent studies [31-33], that have shown an increased scope of central tolerance [34] to self-antigens, our data suggest that the mechanism(s) of natural peripheral tolerance did not evolve the capacity to easily deal with a tissue expressing a multitude of new antigens. We suggest that assessment of tolerance vs. immunity to pre-immunocompetence transplants with graded degrees of antigenic mismatch, could provide a detailed insight into the number of self-antigens peripheral tolerance evolved to handle. While the studies herein suggest that the number lies somewhere between the number of antigens in H-Y mismatched and multiple minor-H mismatched tissue, a more precise definition could be achieved by the stepwise addition of single minor-H mismatches.

The limited capacity of natural peripheral tolerance has important implications for strategies aimed at achieving transplantation tolerance in the clinic. Most basic research towards this goal has been focused on achieving donor specific tolerance by blocking putative 'co-stimulatory' signals. The rationale behind this approach comes from the view that natural self-tolerance involves encounter of self-antigen in the absence of co-stimulatory signals, and that therefore blocking co-stimulatory signals during transplantation should allow natural tolerance mechanisms to take hold. However, our studies with long-healed transplants in immunodeficient recipients, where inflammatory molecules and co-stimulatory triggers are likely to be minimal [2,3,35], suggest that co-stimulation blockade should not work well with strongly mismatched tissue if it really reflects the action of a natural tolerance mechanism. Therefore, since some models using antibody targeting of co-stimulatory molecules, such as CD40L, can induce tolerance to strongly mismatched tissue [36], we suggest that such therapies invoke pathways beyond the natural self-tolerance mechanisms. This view is supported by recent data showing that the ability of targeting CD40L to induce tolerance of donor tissue depends on host mast cells [37]. A similar requirement for NK cells has also been shown [38,39]. Since it would seem unlikely that mast cells or NK cells are required for natural self-tolerance, the data suggest that targeting CD40L not only blocks co-stimulatory signals but also triggers additional regulatory mechanisms unrelated to natural self-tolerance. However, we cannot fully rule out the possibility that long-healed transplants continue to send as yet unrecognized signals that trigger co-stimulation. The data with minor-H mismatches were suggestive of an association between increased graft healing time and natural tolerance. In addition, a small fraction of animals with either MHC or multiple minor mismatched islets in our study appeared tolerant, and this may be due to the increased graft healing time compared to previous studies where tolerance was not observed [18]. More detailed studies will be required to determine the precise contribution of tissue 'health' or homeostasis [2,3,40,41] in any tolerance generated with pre-immunocompetence transplants. Interestingly, we found greater acceptance of MHC mismatched compared to multiple minor-H mismatched pre-immunocompetence islet transplants; a result that is consistent with an increased importance for the indirect pathway in triggering the anti-donor response to long-healed transplants, where a paucity of donor antigen-presenting cells is expected [42-44]. Without donor APCs the direct response is expected to be greatly reduced, leaving T cells triggered via the indirect pathway, that are only able to engage and attack cells of the donor transplant if they are MHC matched.

In summary, our studies provide an explanation for the opposing outcomes in studies of pre-immunocompetence transplants and suggest that systemic donor chimerism is required to obtain consistent natural tolerance to multiple minor-H or MHC mismatched donors.

## Methods

### Mice

Adult wild type C57BL/6, (B6; H-2^b^) BALB/c (H-2^d^) and DBA/2 (H-2^d^), immunodeficient BALB/c-SCID, and day 13 gestation pregnant B6 and BALB/c mice were obtained from NCI (Frederick, MD). Immunodeficient B10.D2-[KO]*Rag2 *(B10.D2-RAG; H-2^d^), B10-RAG and (C57BL/6J × C57BL/10SgSnAi)-[KO]*γc*-[KO]*Rag2 *(hereafter called B10-RAG/γc mice) knockouts were obtained from Taconic Farms (Germantown, NY). Wild type B10.D2 mice were obtained from Jackson Laboratory and BALB.B (H-2^b^) mice from Jackson Laboratory or Taconic Farms. B6.129S7-*Rag1*^*tm1mom *^knockout mice (B6-RAG), originally from Jackson Laboratory were bred on-site at the University of Alberta. All protocols on care and handling of animals were carried out in CCAC or AAALAC accredited facilities.

### Diabetes induction, islet and heart transplantation and nephrectomy

Non-vascularized heart transplantation was done using hearts from <48 hr old neonates. Approximately three quarters of a neonatal heart was grafted either under the skin of the ear pinna or under the renal capsule of recipients. Survival of heart tissue was analyzed by histology and macroscopically by the presence of visible beating for grafts under the renal capsule. In the case of minimally mismatched male heart transplants, while infiltration was observed in some cases (primarily grafts in the ear pinna), the grafts were not rejected under any of the conditions tested. In experiments involving islet transplantation, recipients were generally first made diabetic chemically by a single intraperitoneal injection of streptozotocin (STZ; 200 mg/kg for B6-RAG and 210 mg/kg for BALB/c-SCID). Diabetes was confirmed by measuring blood glucose (>15.0 mmol/L; most recipients were >20.0 mmol/L). Approximately 500 donor islets were placed into the renal subcapsular space of diabetic, and, in a few cases in single minor-H mismatched transplants, non-diabetic recipients (to exclude any confounding effects on tolerance or immunity from STZ- and/or diabetes-related immunosuppression). Where indicated, second islet transplants were performed in recipients with an intact first islet transplant (acceptance >120 days), in the contralateral kidney. Nephrectomy was done at least 10 days post second transplant to remove the kidney bearing first set islets and assess second set islet survival. Graft rejection was defined as two consecutive blood glucose measurements >15.0 mmol/L taken on two different days.

### Immune reconstitution of immunodeficient recipients of transplants

Fetal livers were extracted from the fetuses of day 14 or 15 gestation pregnant female mice, pooled and homogenized into a single cell suspension, counted and resuspended in PBS before injection into immunodeficient recipients. For reconstitution of BALB/c-SCID and B6-RAG mice, 20–40 and 15–30 million cells respectively were injected intravenously. For H-Y mismatch experiments and reconstitution of female RAG mice, each fetus was sex-determined by PCR or when possible by visual identification of sex organs under a dissecting microscope and only female fetuses were pooled for use. Detection of an amplicon of the Sry gene found on the Y chromosome (primer sequences: forward 5'-AGACAAGTTTTGGGACTGGTGAC-3' and reverse 5'-AGCCCTCCGATGAGGCTGATA-3'), employed standard PCR conditions.

### Flow cytometry, peritoneal cells, T cell and NK cell depletion

Fluorescent anti-mouse pan-TCRβ, CD19, and CD8α antibodies were purchased from eBioscience (San Diego, CA), anti-mouse CD4 from Caltag (Burlingame, CA), and anti-mouse Ly-9.1, CD5, CD5.1, CD43, H-2D^d^, H-2D^b^, Vβ6 and Vβ8 were from BD Pharmingen (San Diego, CA). Cells were first incubated with a cocktail consisting anti-CD16/32 antibody (2.4G2; Bio Express, West Lebanon, NH), and mouse, rat and hamster sera to prevent binding via the Fc portion. Four-color antibody staining was then carried out simultaneously by incubating cells with the appropriate antibodies at 4°C for 15 minutes, washing and resuspending in PBS prior to flow cytometry. Red blood cells were lysed when staining PBMCs. Where biotinylated antibodies were used, secondary incubation with allophycocyanin-conjugated streptavidin (eBioscience) was performed and followed by a second washing before analysis. A FACSCalibur™ flow cytometer (BD Biosciences) equipped with CellQuest™ Pro software was used for data acquisition and analysis. NK cells were depleted from B6-RAG mice by i.p. injection of 35 μl of anti-asialo GM1 antibody (Wako Chemicals USA, Richmond, VA) 10 and 3 days prior to injection of donor cells. Depletion of NK cells was assessed by staining with antibody to NK1.1 (PK136; eBioscience). T cells were depleted from peritoneal lavage cell preparations using anti-CD90 microbeads and MACS™ cell separation columns according to the manufacturer's instructions (Miltenyi Biotec, Auburn, CA). As measured by flow cytometry, the peritoneal cell preparations contained approximately 1% T cells after the depletion procedure; 2 × 10^6 ^donor peritoneal cells were injected i.v. or i.p. into recipients.

### CTL assay

CTL responses were assayed by the JAM Test as described [45]. Briefly, recipient splenocyte responder cells were stimulated for 6 days with irradiated donor or 3^rd ^party splenocytes. Targets were ^3^H-thymidine labeled Con-A stimulated splenocyte blasts. Killing of targets was tested at various responder to target ratios and after killing of control syngeneic targets was subtracted, donor specific killing was expressed as lytic units calculated as the dilution of responders giving 20% killing of targets.

### Histology

Graft bearing kidneys from recipient mice were fixed in formalin, embedded in paraffin, sectioned, and stained with hematoxylin and eosin.

### Statistical analysis

Statistical analyses included the Kaplan-Meier method and the log rank test for graft survival data, the Kruskal-Wallis test followed by Dunn's multiple comparison test for CTL responses and donor peritoneal cell chimerism, and a one-way ANOVA and Tukey's multiple comparison test for TCR-Vβ expression (GraphPad Prism Software, San Diego, CA).

## Abbreviations

FL, fetal liver; Tx, transplant; PBL, peripheral blood lymphocytes; minor-H, minor histocompatibility; KO, knockout; WT, wild type

## Competing interests

The author(s) declare that they have no competing interests.

## Authors' contributions

WFNC designed and carried out the islet transplantation studies and drafted the manuscript. AP-D contributed to the design and data generation in heart graft experiments and revised the manuscript. HR contributed data on peritoneal cell chimerism and revised the manuscript. CCA conceived and designed the study, carried out the heart transplant studies, and drafted the manuscript.

## Reviewers' comments

### Reviewer's report 1

Dr. Matthias von Herrath. La Jolla Institute for Allergy and Immunology, San Diego CA, United States.

In this manuscript the authors study whether the ability of natural tolerance to allogeneic tissue might be determined by the degree of antigenic mismatch, by the type of tissue or its location. By using mismatched tissue transplants the authors show that natural tolerance to allogeneic tissue requires chimerism to be fully functional. Moreover, they show that natural tolerance to minimally mismatched heart or islet tissue will be established when transplanted 'internally' but not in the skin. The manuscript is interesting and the conclusions correct. However, a few specific points have to be addressed, which are listed below.

1. The authors describe that neonatal heart grafts in the BALB to B6 combination generated B cell chimerism while islet transplants generated almost exclusively T cell chimerism. These two populations should be studied in more detail and the following questions could be addressed: Which cytokines are produced by these 'chimeric' B or T cells? Are these cells activated and responsive to heart or islet antigens respectively? Do these cells (chimeric B or T cells) prolong graft survival when transferred into recipient animals post heart or islet transplant respectively?

#### Response

We agree that it would be of interest to determine which cytokines, if any, are produced by the passenger B and T cells that generate chimerism, as this may influence the mechanism of tolerance induced. This is an aspect we will dissect in future studies. For the current manuscript our main conclusion regarding chimerism is that the overall outcome is the same (tolerance of the donor transplant) whether the chimerism detected is primarily B cell chimerism or T cell chimerism. We do find that the passenger T cells appear to have been activated (CD44^hi^, relative to a naïve 'normal' T cell population; data not shown). This is similar to our previous work [20] where we found that passenger T cells that migrated out of skin grafts were oligoclonal and of a memory phenotype and it would certainly be of interest to determine if either the passenger B or T cells have a high proportion of cells with antigen receptors specific to antigens found in the tissues transplanted (heart, islets or skin). However, we do not consider it likely that these passenger cells have a strong regulatory capacity capable of actively blocking rejection of the tissue transplant. This view is based on the observation that the chimerism from passenger cells in islets only prevents rejection when the passenger cells are present before immune system generation. In contrast, we find they enhance rejection of the transplant when the islet transplant is given after the generation of immunocompetence (our unpublished data). Therefore, we consider that the primary role of the passenger cells is to serve as a source of antigen, antigen that leads to tolerance in a newly generated immune system and immunity in an already mature immune system.

2. Figure 2C presents histology of a post-FL BALB.B heart graft with infiltrating cells. What is the nature of these cells? Immunohistochemistry could be performed to detect CD4, CD8 and/or B cells.

#### Response

While we have not yet analyzed the type of cells infiltrating/rejecting the graft, we expect the infiltration would include T cells and/or macrophages, as we are unaware of any examples of tissue rejection mediated by B cells, rather than T cells, when the only mismatches are minor histocompatibility antigens.

### Reviewer's report 2

Dr. Irun R. Cohen. Department of Immunology, The Weizmann Institute of Science, Rehovot

Director, National Institute for Biotechnology in the Negev Ben-Gurion University of the Negev Beer-Sheva, Israel

Major comments:

1. The paper deals with a very important question and examines it in a critical, in-depth fashion. This study, to my mind, is a valuable contribution, and should be published.

2. The results, however, could be made more comprehensible by redoing the figures. I had to invest time and effort to understand most of the figures. The reader should not have to work to get the message; the authors should make it easy for the reader to grasp the point. A reader who has to keep consulting the legend and the text to follow the point of a figure will put the paper aside unread.

3. The title is not really clear.

4. Statistical significance needs to be added to many of the figures.

5. The paper uses a number of key terms that are not defined, or are defined only in passing or late in the paper. These terms are important to the conclusions. Unfortunately, different immunologists define them differently according to their preferred operational, mechanistic, or molecular criteria. I suggest that it would be helpful to have these key terms defined at the outset so that the reader will know how the authors see the matter. The terms are: Tolerance; natural tolerance; central tolerance; peripheral tolerance; and ignorance.

6. The text is a bit wordy and redundant in some places.

#### Response

We agree it is critical to make the message of the data easy to follow and to facilitate this we have added, to some of the figures, diagrams that describe the experiment. We have added an 'Additional File 1' referred to in the introduction, that provides our definition of the important terms and removed text when it is redundant. We expanded the title to make it less cryptic, and statistical analyses have been added to appropriate figures.

7. The results demonstrate the association of stable chimerism with acquired non-reactivity to the allograft. Is the chimerism the cause of the non-reactivity, or is it merely the expression of non-reactivity? How might chimerism induce non-reactivity?

#### Response

Since grafts lacking passenger lymphocytes were not tolerated (i.e. were rejected), grafts containing passenger lymphocytes were accepted, and lymphocytes alone were accepted (as long as they were systemic) we consider the simplest interpretation is that the lymphocytes generate the tolerant state and are not the consequence of such a state. To more greatly solidify this interpretation it would be ideal to show that elimination of the chimerism abrogates tolerance, and Bonilla et al. recently confirmed this prediction [46]. However, we have not embarked on such experiments, in our model, as it is difficult to eliminate chimerism in an easily interpretable fashion. For example, if chimerism is eliminated by antibody treatment or transfer of CTL specific to chimeric cells, is the resulting abrogation of tolerance due to elimination of chimerism or generation of an inflammatory milieu (e.g. immune complexes in the case of antibody depletion)?

8. I recall that some papers published in the 60s or 70s of the last Century reported rejection of syngeneic skin grafts (Mariani T, Dent PB, Good RA. Alteration of skin in gross leukemia. I. Syngeneic skin-graft rejection and tumor development. J Natl Cancer Inst. 1970 Feb;44(2):319-28, and others). Do the data reported in this paper have any bearing on such old observations?

#### Response

Rejection of sygeneic grafts or modified syngeneic grafts (haptenated or tumor bearing) can occur (examples: [47-50]). In contrast, our previous work [2] showed a lack of spontaneous or graft induced autoimmunity in a stem cell reconstitution model, and we did not see infiltration of host tissues (ovaries, thyroid/parathyroid, skin, kidney, pancreas, heart, lung, liver, small intestine; not shown) or rejection of syngeneic islets (Figure 3) in the current study. We speculate that the main difference between our studies and these earlier ones, is that we have examined the response to unmodified syngeneic tissue that has healed-in prior to immune system generation. However, while we do not have evidence of attack on syngeneic tissue, we consider that our model has the potential to show rejection of syngeneic tissue grafts that are not well healed in. In a model of recipient reconstitution with mature T cells, rejection of syngeneic skin grafts (not healed in) has been observed [48]. The stem cell reconstitution model we have used, similar to an elegant but more difficult model of McCullagh [49], should be a fruitful tool to determine which factors are able to prevent the establishment of self tolerance to particular tissues.

### Reviewer's report 3

Dr. Wei-Ping Min. Departments of Surgery, Pathology, Microbiology & Immunology and Oncology, University of Western Ontario, London, Ontario, Canada (nominated by David Scott, University of Maryland School of Medicine Center for Vascular and Inflammatory Diseases, Baltimore MD, United States)

Comments:

1) This paper addresses immune tolerance and ignorance by using immune deficient Rag-/- mice before and after reconstitution of immune system with liver hematopoietic stem cell. Donor tissues survived after being placed under the kidney capsule, but were rejected when placed under the skin in a one Ag mismatch model. The author claims immune tolerance is responsible for the observed results rather then immune ignorance, since CTL responses were observed in this model.

In the 2nd part of the results, the author further addressed a similar mechanism using a multiple minor Ag mismatch model. The results indicated that passenger lymphocytes are critical in establishing chimerism and tolerance after transplantation.

There are several unclear aspects in these results. First, the authors did not clearly explain the advantage of using a non-vascularized heart graft rather than a normal heterotopic vascularized heart for transplantation. It is interesting to see that a non-vascularized graft can last in recipients for long periods of time, which may produce a convenient model for transplantation studies.

The author also concluded that the location of the graft, whether it is placed under the skin or under the kidney capsule, is important in priming for tolerance, which has been well-documented in transplantation. A discussion is needed for this part.

#### Response

We agree that the non-vascularized graft could be a convenient model for transplantation studies and it has been used extensively for heart grafts in the ear pinna. We used the non-vascularized heart model because it allowed us to more easily compare different sites of transplantation (ear vs. kidney) and compare different tissues in the same site (islets vs. heart placed in the kidney). We have stated this more clearly in the updated manuscript. We have added a discussion of some of the literature defining the importance of graft location in priming vs. 'tolerance', an issue that relates to sites considered 'immune privileged'. The sub-renal capsule location is not considered immune privileged, as grafts placed there, in immune competent animals, normally trigger immune responses and rejection. In addition, other than our studies presented in Figure 1, we have not found any previous study showing that a specific tissue primes immunity when transplanted under the skin but instead induces tolerance when placed under the kidney capsule.

2) CTL responses were observed after reconstitution with fetal liver hematopoietic stem cells, which could not mediate graft rejection. In this part it is important to show efficacy of the reconstitution by increasing the dose of cells.

#### Response

The lack of rejection of the male grafts shown in Figure 1 is not an indication of poor immune reconstitution. Even wild type B6 female mice do not reject male heart (our unpublished data) or islet transplants [51]. In a separate set of B6-RAG-KO female recipients, not shown in the manuscript, we confirmed our earlier data [2] that the immune reconstitution with fetal liver cells is sufficient to restore the ability to reject even weakly mismatched (male) established skin grafts (skin grafts given pre-fetal liver). Together with the strong CTL responses to H-Y in control recipients given female hearts, reconstituted at the same age, and immunized with male spleen cells post fetal liver reconstitution (Figure 1), we consider these data strong evidence for good immune reconstitution.

3) In the 3rd part of the results, the author concluded that NK cells could mediate cellular graft rejection systematically but not locally (peritoneal cavity). Donor cells, injected either i.v. or i.p., were eliminated in the spleen but not in the peritoneum. However, in the second part of the results, the data indicated that passenger cells reconstituted the recipient and established chimerism and tolerance. The author should have a section discussing this difference, and explaining how passenger cells escape NK cell responses systematically in the second part of the results.

4) The rationale between the second and third part of the results should be elaborated in greater detail.

#### Response

We have added a brief discussion of loss vs. acceptance of systemic donor cells that is controlled by NK cells. We consider that the tolerance of donor cells, even after a fetal liver inoculum that reconstitutes the NK cell lineage, would occur because the NK cells develop in the presence of the donor cells and adjust their activating and inhibitory receptors accordingly. The opportunity to make this developmental alteration in receptors is not present for an already fully developed NK cell repertoire in the RAG-KO recipient. We have also added more description in the results section to explain the link between the second and third part of the results.

## Supplementary Material

Additional File 1 Definition of key terms. Click here for file

Additional File 2 Pre-immunocompetence male islet transplants are not ignored but instead induce tolerance in the newly generated recipient immune system. Diabetic B6-RAG female recipients were given a B6-RAG male islet transplant followed 2 days (n = 3) or 8–10 wks later (n = 7) by an injection of female B6 fetal liver (FL) cells; these were grouped together as no difference in CTL for the islet transplant recipients was observed between these different healing times. Three months after FL cell injection (after immune system generation in the recipients) the mice were immunized i.p. with 5 × 10^6 ^irradiated (15 Gy) wild type male B6 spleen cells in PBS. A further 3 months later, all animals were immunized i.p. with 5 × 10^6 ^irradiated or non-irradiated wild type BALB.B spleen cells in PBS. Three to 5 weeks later, spleens were removed and 6 × 10^6 ^recipient splenocytes were cultured with either irradiated B6 male or BALB.B third party stimulator cells and assayed for killing of B6 male vs. female and BALB.B targets. For comparison of H-Y and BALB.B specific killing, maximum % killing after subtraction of killing of syngeneic targets is shown for individual animals, along with regression lines and 95% confidence intervals. Recipients of male islet transplants appeared specifically hyporesponsive to H-Y, but killed BALB.B control targets (r = -0.082), in contrast to control mice lacking male islet transplants that demonstrated a correlation between the ability to kill both BALB.B and H-Y targets (r = 0.823). Controls include recipients with a female islet transplant and non-transplanted B6 females. Click here for file
