# Supplementary material for: The ability of natural tolerance to be applied to allogeneic tissue: determinants and limits
Source: Biol Direct. 2007 Apr 16;2:10. doi: 10.1186/1745-6150-2-10 (PMC1854886; doi:10.1186/1745-6150-2-10)
Supplement: Additional File 1 — Definition of key terms. [file 1745-6150-2-10-S1.pdf]

Additional File 1

Definition of key terms:

Tolerance: Can generally be defined as an absence of immunity towards what would otherwise be an immunogenic antigen challenge, and results from prior exposure to the specific antigen(s). Tolerance can therefore be considered a negative response to antigen. The existence of a state of tolerance may either be directly tested by experimental challenge with an immunogen, or inferred by survival or persistence of otherwise immunogenic antigen(s) in the putatively tolerant host.

Natural tolerance: Tolerance that is induced solely by naturally evolved mechanisms involved in generating self tolerance, and not including manipulation of immunity by introduction of pharmacologic agents or antibodies that may invoke distinct mechanisms.

Central tolerance: Antigen specific unresponsiveness due to recognition of antigen by lymphocyte precursors during the process of lymphocyte development. Central tolerance is a result of antigen encounter before lymphocytes have matured to the point where they are able to respond in a positive fashion (immunity) to antigen. This process occurs primarily in the bone marrow for B cells and in the thymus for T cells, but may extend beyond these sites if lymphocyte maturation is not complete prior to migration into the 'periphery'.

Peripheral tolerance: Antigen specific unresponsiveness due to recognition of antigen by fully mature lymphocytes (i.e. lymphocytes that have the potential to respond positively to antigen) and not due to encounter with antigen during lymphocyte development. This process could occur in any tissue (including antigen encounter in the thymus by re-circulating mature T cells).

Ignorance: A state where lymphocytes in an animal appear neither to be tolerant of, nor immunized (primed) to, an antigen present in the animal; the animal's immune system appears 'naïve' despite the presence of antigen. Ignorance could truly reflect a naïve state of all relevant specific lymphocytes or instead the sum total of positive and negative interactions of individual lymphocytes with antigen, giving the overall appearance of a naïve state (a zero sum).
