# Supplementary figures and images for: The ability of natural tolerance to be applied to allogeneic tissue: determinants and limits
Source: Biol Direct. 2007 Apr 16;2:10. doi: 10.1186/1745-6150-2-10 (PMC1854886; doi:10.1186/1745-6150-2-10)

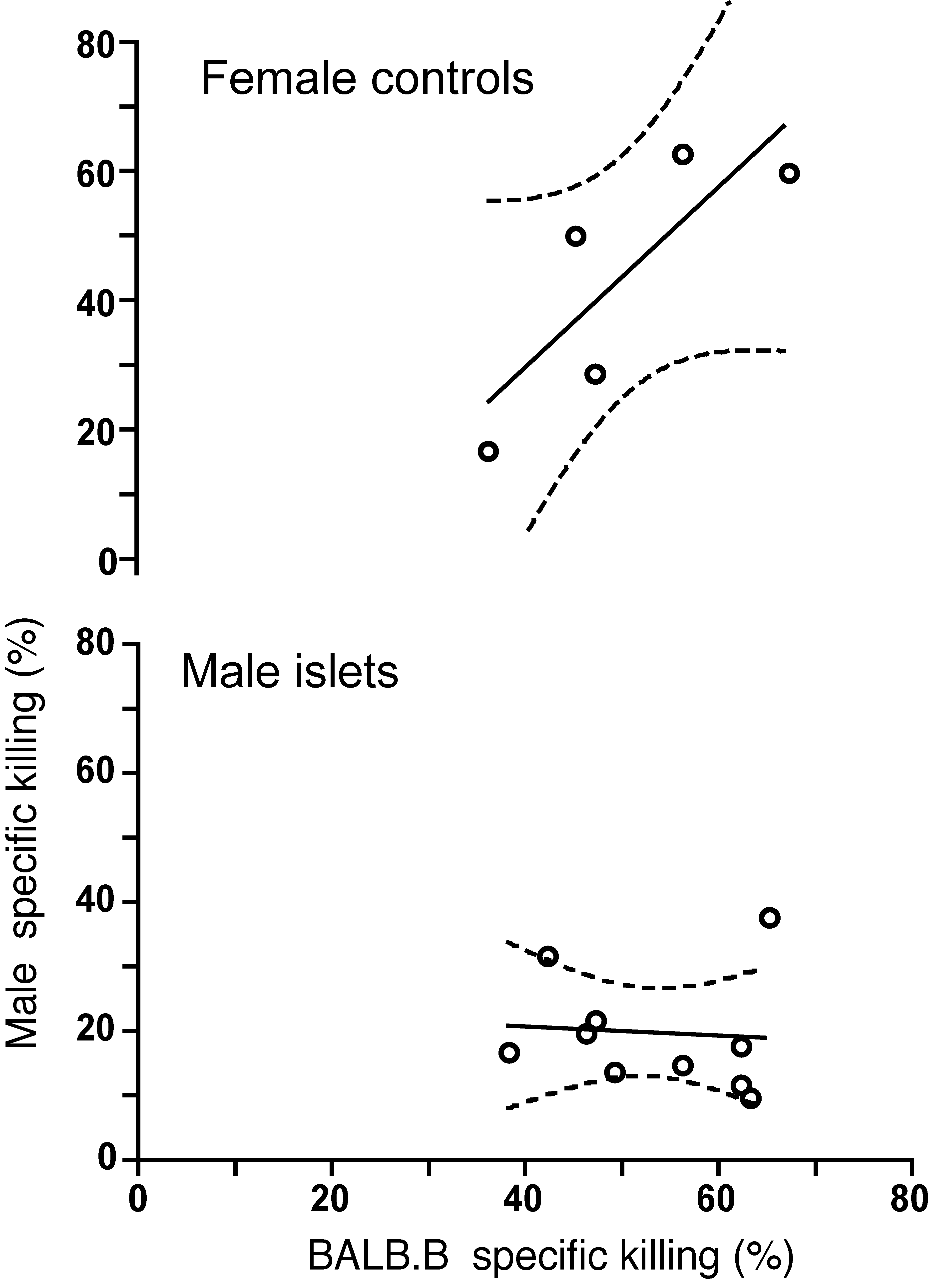

Supplement: Additional File 2 — Pre-immunocompetence male islet transplants are not ignored but instead induce tolerance in the newly generated recipient immune system. Diabetic B6-RAG female recipients were given a B6-RAG male islet transplant followed 2 days (n = 3) or 8–10 wks later (n = 7) by an injection of female B6 fetal liver (FL) cells; these were grouped together as no difference in CTL for the islet transplant recipients was observed between these different healing times. Three months after FL cell injection (after immune system generation in the recipients) the mice were immunized i.p. with 5 × 10^6^ irradiated (15 Gy) wild type male B6 spleen cells in PBS. A further 3 months later, all animals were immunized i.p. with 5 × 10^6^ irradiated or non-irradiated wild type BALB.B spleen cells in PBS. Three to 5 weeks later, spleens were removed and 6 × 10^6^ recipient splenocytes were cultured with either irradiated B6 male or BALB.B third party stimulator cells and assayed for killing of B6 male vs. female and BALB.B targets. For comparison of H-Y and BALB.B specific killing, maximum % killing after subtraction of killing of syngeneic targets is shown for individual animals, along with regression lines and 95% confidence intervals. Recipients of male islet transplants appeared specifically hyporesponsive to H-Y, but killed BALB.B control targets (r = -0.082), in contrast to control mice lacking male islet transplants that demonstrated a correlation between the ability to kill both BALB.B and H-Y targets (r = 0.823). Controls include recipients with a female islet transplant and non-transplanted B6 females. [file 1745-6150-2-10-S2.tiff]
